# Animal Venoms as Potential Antitumor Agents Against Leukemia and Lymphoma

**DOI:** 10.3390/cancers17142331

**Published:** 2025-07-14

**Authors:** Geovanna M. Malachias-Pires, Eloise T. M. Filardi, Marcela Romanazzi, Julia Lopes-de-Oliveira, Isabela C. dos Santos, Guilherme Melo-dos-Santos, Ana Beatriz Rossi, Michele Procópio Machado, Thiago A. da Silva, Manuela B. Pucca

**Affiliations:** 1 Graduate Program in Bioscience and Biotechonology Applied to Pharmacy, School of Pharmaceutical Sciences, São Paulo State University (UNESP), Araraquara 19060-900, São Paulo, Brazil; geovanna.malachias@unesp.br (G.M.M.-P.); e.filardi@unesp.br (E.T.M.F.); marcela.romanazzi@unesp.br (M.R.); isabela.caroline@unesp.br (I.C.d.S.); guilherme.melo-santos@unesp.br (G.M.-d.-S.); michele.machado@usp.br (M.P.M.); thiago-aparecido.silva@unesp.br (T.A.d.S.); 2 Department of Clinical Analysis, School of Pharmaceutical Sciences, São Paulo State University (UNESP), Araraquara 14800-903, São Paulo, Brazil; julia.lopes-oliveira@unesp.br (J.L.-d.-O.); anabeatrizrossimed@gmail.com (A.B.R.)

**Keywords:** hematological malignancies, cytotoxicity, apoptosis, L-amino acid oxidase (LAAO), phospholipase A_2_ (PLA_2_), melittin, anticancer therapy

## Abstract

Leukemias and lymphomas are hematologic malignancies characterized by complex pathophysiological mechanisms and increasing global incidence. Despite advances in chemotherapy, immunotherapy, and targeted therapies, challenges such as drug resistance and relapse persist, necessitating novel therapeutic strategies. This review explores the cytotoxic potential of venoms derived from snakes, bees, and scorpions against leukemia and lymphoma cells. Numerous venom-derived components, such as L-amino acid oxidases (LAAOs), phospholipases A_2_ (PLA_2_s), and peptides like melittin, demonstrate selective antitumor activity through mechanisms involving oxidative stress, apoptosis induction, cell cycle arrest, and immunomodulation. These molecules exert their effects via mitochondrial pathways, caspase activation, and inhibition of pro-survival signaling cascades such as NF-κB and PI3K/Akt. Despite promising preclinical results, the clinical translation of these bioactive compounds remains limited due to challenges in standardization, delivery, and safety profiling. This review highlights recent advances in venom research, summarizes key molecular targets, and discusses future directions to harness venom-derived molecules as innovative therapies for hematological cancers.

## Simple Summary

Leukemia and lymphoma are types of blood cancer that affect many people worldwide and often have limited treatment options, especially when the disease returns after therapy. In recent years, scientists have explored natural sources to discover new ways to fight these cancers. Animal venoms—such as those from snakes, bees, and scorpions—contain substances that can eliminate cancer cells or prevent them from multiplying. This review examines how certain venom components act on leukemia and lymphoma cells in laboratory studies. These substances can cause cancer cells to lose their function, stop dividing, or undergo controlled cell death. Some venom molecules may also help the immune system recognize and attack cancer cells. Although these findings are mostly from laboratory research and are not yet used in standard medical treatments, they offer promising ideas for developing new medicines. Understanding how these venoms work may lead to therapies that are more effective and have fewer side effects. This work highlights the potential of nature-inspired solutions in the fight against cancer and opens the door to future research that could benefit patients around the world.

## 1. Introduction

Venom from venomous animals has emerged as a promising source of interest in medical research, driven by its remarkable therapeutic potential. Throughout history, snake venom, for instance, has been used in various cultures for medicinal purposes [1]. This ancestral use suggests an empirical foundation for modern investigations, indicating that the therapeutic potential of these venoms is not an entirely new discovery, but rather a scientific rediscovery and validation of traditional practices [2]. Recent reviews and studies have consistently highlighted the broad therapeutic potential of animal venoms for a wide range of medical conditions, including arthritis, asthma, diabetes, and neurodegenerative diseases [3,4]. This broad pharmacological activity suggests that venoms contain a diverse array of bioactive molecules capable of interacting with multiple biological pathways, thereby increasing the likelihood of identifying effective compounds against cancer [5]. Given the growing resistance of cancer cells to conventional chemotherapeutic agents, it becomes imperative to explore alternative therapeutic strategies. In this context, animal venoms represent a promising avenue for investigation, due to the specific and potent nature of their components, which may offer mechanisms of action capable of overcoming drug resistance in cancer cells [6].

A variety of snake, spider, scorpion, and other venomous animal species are capable of producing bioactive substances that exhibit significant effects on cancer cells [7]. Venoms are complex mixtures composed of proteins, peptides, enzymes, and toxins that can interfere with critical cellular processes [8]. These substances may act by inhibiting protein synthesis and inducing processes such as angiogenesis and apoptosis [9]. The bioactive peptides present in venoms can also influence key cancer hallmarks, such as cell proliferation, invasion, and migration, as well as modulate the immune response [10]. Among the bioactive components identified in snake venoms are proteolytic enzymes, arginine ester hydrolases, thrombin, thrombin-like enzymes, collagenase, and hyaluronidase [11].

One of the main advantages of venom-derived compounds, compared to traditional chemotherapy, lies in their ability to specifically target cancer cells with lower toxicity to normal tissues. Chemotherapy, due to its generalized cytotoxicity, often causes severe side effects. The molecular diversity found in venoms also allows for multiple mechanisms of action against cancer cells, which may be crucial in overcoming resistance mechanisms that tumor cells develop against therapies targeting a single pathway [12].

Leukemias and lymphomas represent aggressive types of cancer that affect the hematopoietic and lymphatic systems, respectively. These diseases pose a significant challenge in oncology due to their invasive nature and high recurrence rates [13]. Although cancer mortality rates have declined in recent years, the incidence of leukemia and other malignancies has been increasing [14]. The treatment of these neoplasms is often hindered by high initial costs, limited access to advanced therapies, significant side effects, the development of drug resistance, and frequent relapses [15]. The rising incidence of leukemia and lymphoma, despite therapeutic advances, highlights the ongoing need for more innovative and effective treatment strategies—such as those potentially offered by venom-derived compounds [16]. The specific challenges associated with the treatment of hematological cancers, including drug resistance and recurrence, may be overcome by the complex and diverse mechanisms of action exhibited by venom components. The multifaceted nature of venom effects on cancer cells may bypass specific resistance mechanisms that tumor cells develop against single-target therapies [17].

The ability of venom components to induce cytotoxicity, promote apoptosis, and inhibit cell proliferation opens new perspectives for the development of more effective and targeted therapies [18]. Studies have investigated the application of venoms from different species, evaluating their effects on tumor cell cultures and animal models [19].

Promising results have demonstrated that venom components can trigger programmed cell death (apoptosis), disrupt the cell cycle, cause DNA damage, and reduce cell viability [20]. Their mechanisms of action include the disruption of the cell membrane, interaction with membrane phospholipids and carbohydrates, modulation of ion channels and receptors, and interference with intracellular signaling cascades [21]. The consistent observation of venom components inducing apoptosis and inhibiting cell proliferation across various cancer cell lines provides robust preliminary evidence for their anticancer potential [22].

This literature review aims to compile and analyze the available scientific evidence regarding the cytotoxic effects of venoms from venomous animals on leukemia and lymphoma tumor cells by providing a detailed analysis of the known mechanisms of action, present the results of laboratory studies, and discuss the potential clinical implications of these findings.

Considering the significant biological, pathological, and therapeutic differences between hematological malignancies and solid tumors, this review intentionally focuses exclusively on leukemia and lymphoma. Unlike solid tumors, which present as localized masses, hematological cancers involve disseminated malignant cells in the bloodstream and lymphatic tissues, leading to unique disease dynamics and therapeutic challenges. Furthermore, venom components may interact differently with hematological versus solid tumor microenvironments, influencing their mechanisms of action, pharmacokinetics, and toxicity profiles. The existing literature often analyzes venom-derived compounds across both tumor types indiscriminately, potentially obscuring critical distinctions in efficacy and safety. By restricting our analysis to non-solid tumors, this review aims to provide a more targeted, clinically relevant, and biologically coherent assessment of animal-venom-derived agents in the context of leukemia and lymphoma.

## 2. Materials and Methods

A systematic literature search was conducted to identify studies evaluating the cytotoxic effects of animal venoms and their components on leukemia and lymphoma cells. Searches were performed in the electronic databases PubMed, MEDLINE, Scopus, and Web of Science, including all articles published up to May 2025. The search strategy combined relevant keywords such as “animal venom,” “cytotoxicity,” “leukemia,” “lymphoma,” “venom components,” and “anticancer activity.” Studies were screened based on predefined inclusion and exclusion criteria. Inclusion criteria comprised original research articles, reviews, and clinical studies that investigated animal venom components with demonstrated activity against hematological malignancies, particularly those offering mechanistic insights or assessing therapeutic potential. Exclusion criteria encompassed studies unrelated to leukemia or lymphoma, those lacking sufficient experimental detail or clinical relevance, and non-peer-reviewed sources (e.g., conference abstracts, editorials, and commentaries). Full texts of potentially eligible studies were assessed for inclusion by two independent reviewers, with discrepancies resolved by discussion or consultation with a third reviewer. Data extraction included details on venom source, target cell lines, observed cytotoxic effects, proposed mechanisms of action, and potential therapeutic applications. Extracted data were synthesized qualitatively to provide a comprehensive overview of current evidence, highlighting key venom components with anticancer potential and identifying gaps for future research.

## 3. Leukemia and Lymphoma: Basic Principles

Leukemia and lymphoma represent a diverse group of hematological malignancies arising from hematopoietic and lymphoid cells, respectively. Their classification is based on several factors, including cell lineage (myeloid vs. lymphoid), disease progression (acute vs. chronic), cellular maturity, and distinct pathological features such as Hodgkin vs. non-Hodgkin lymphoma, all of which are crucial for understanding disease biology and guiding clinical management [23].

Recent data from GLOBOCAN 2022 highlight the substantial global burden of these diseases. Together, leukemia and lymphoma accounted for approximately 1.12 million new cases and 578,000 deaths worldwide in 2022. Non-Hodgkin lymphoma (NHL) remains the most frequently diagnosed among the three, followed by leukemia and then Hodgkin lymphoma (HL). However, leukemia presents higher mortality rates globally, reflecting its aggressive nature and the challenges associated with its treatment. Geographic patterns show higher incidence rates in developed countries, while the largest absolute number of cases occurs in densely populated regions, such as Asia. Disparities in healthcare infrastructure further contribute to higher mortality, especially for HL in low-resource settings [24].

From a biological perspective, leukemias result from genetic and epigenetic alterations affecting hematopoietic stem and progenitor cells within the bone marrow, disrupting normal differentiation pathways and leading to the accumulation of immature, malignant cells in the bone marrow, bloodstream, and peripheral tissues [25,26].

Lymphomas, on the other hand, originate from lymphoid cells located in secondary lymphoid organs, such as lymph nodes, spleen, and mucosa-associated lymphoid tissues (MALT). Under pathological conditions, genetic mutations and chronic antigenic stimulation may drive the uncontrolled proliferation and survival of malignant lymphoid clones, contributing to tumor formation [27].

Lymphomas are broadly classified into Hodgkin lymphoma (HL), accounting for approximately 10% of cases, and non-Hodgkin lymphomas (NHLs), which represent the remaining 90% [28]. HL is characterized by the presence of Hodgkin and Reed–Sternberg (HRS) cells, typically derived from B lymphocytes and often associated with Epstein–Barr virus (EBV) infection. HL is further subdivided into classical HL (cHL)—with four histological subtypes, the most common being nodular sclerosis—and nodular lymphocyte-predominant HL (NLPHL), a rarer form with an indolent course [29,30]. In contrast, NHLs encompass a heterogeneous group of malignancies classified according to the cell of origin (B-cell, T-cell, or NK-cell neoplasms). Clinically, NHLs are also divided into aggressive (high-grade) and indolent (low-grade) subtypes, a distinction that directly influences treatment decisions and prognosis [31,32].

Leukemia arises from genetic and epigenetic alterations in hematopoietic progenitor or precursor cells within the bone marrow, leading to impaired differentiation and the accumulation of immature malignant cells in the bone marrow, bloodstream, and other tissues [33,34]. These malignancies present a wide spectrum of clinical and molecular subtypes, primarily affecting white blood cells, and are often associated with environmental risk factors such as ionizing radiation, toxic chemicals, infections, and socioeconomic conditions that may influence disease pathogenesis. Environmental factors such as smoking, chronic stress, physical debilitation, frequent exposure to toxic substances, and susceptibility to viral infections may increase the risk of developing leukemia, especially in individuals with a genetic predisposition or unhealthy lifestyle habits [35]. Leukemias are broadly classified into four major subtypes based on cell lineage (myeloid or lymphoid) and disease progression (acute or chronic): acute myeloid leukemia (AML), chronic myeloid leukemia (CML), acute lymphoblastic leukemia (ALL), and chronic lymphocytic leukemia (CLL) [36,37].

Acute myeloid leukemia (AML) is an aggressive cancer characterized by the accumulation of immature myeloid cells, leading to hematopoietic failure and increased risk of infections, bleeding, and anemia [38]. In contrast, chronic myeloid leukemia (CML) progresses more slowly and is marked by the presence of the Philadelphia chromosome (BCR-ABL fusion gene), which promotes the uncontrolled proliferation of myeloid cells [39].

Acute lymphoblastic leukemia (ALL), more frequent in children, is characterized by the excessive proliferation of immature B and T lymphoblasts, which infiltrate the bone marrow, blood, and other tissues [40]. Chronic lymphocytic leukemia (CLL) predominantly affects older adults and involves the gradual accumulation of dysfunctional B lymphocytes, often with an asymptomatic course in early stages. Both subtypes—acute myeloid leukemia (AML), chronic myeloid leukemia (CML), acute lymphoblastic leukemia (ALL), and chronic lymphocytic leukemia (CLL)—present distinct progression patterns, clinical features, and therapeutic responses, reflecting the biological and clinical complexity of leukemia [41].

An important subtype of acute leukemia is the MLL1-rearranged leukemia (MLL1r), characterized by chromosomal translocations involving the MLL1 (KMT2A) gene. This subtype is commonly observed in pediatric and infant acute leukemias and is associated with poor prognosis and aggressive clinical behavior [42]. MLL1r leukemias are driven by fusion oncoproteins that alter transcriptional programs and promote leukemogenesis by dysregulating genes involved in hematopoietic development and differentiation. Current research focuses on developing therapies that target critical protein–protein interactions within these fusion complexes [43].

### Therapeutic Strategies Available for Leukemia and Lymphoma

**Figure 1:**
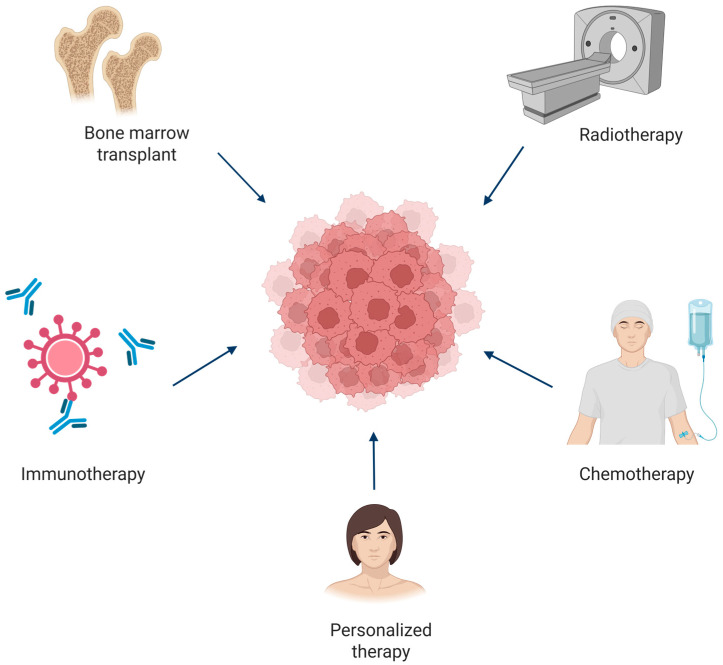
Available treatments for leukemia and lymphoma. Created with Biorender.com.

Currently, therapeutic approaches for the treatment of lymphomas and leukemias share notable similarities. These include chemotherapy, which employs cytotoxic agents to destroy malignant cells, and immunotherapy, which enhances the patient’s immune system to recognize and eliminate cancer cells [44]. Targeted therapies have also been applied, which act on specific molecular targets associated with cancer cell growth and survival, and hematopoietic stem cell transplantation (HSCT), which replaces damaged or diseased bone marrow with healthy progenitor cells to reestablish normal hematopoiesis [45] (Figure 1 and Table 1). These strategies can be employed individually or in combination, depending on the cancer type, stage, and the patient’s overall health status, with the goal of maximizing treatment efficacy and enhancing quality of life [46].

In the realm of leukemia, targeted therapy has emerged as a particularly effective strategy in its management, providing a level of therapeutic precision that significantly reduces the characteristic adverse effects of conventional chemotherapy. A notable milestone in this field was the development of imatinib, a tyrosine kinase inhibitor that revolutionized the management of chronic myeloid leukemia (CML) by specifically targeting the BCR-ABL protein responsible for the pathology [47]. Clinical studies have demonstrated that imatinib results in high rates of molecular response and prolonged remission in CML patients, representing a remarkable advancement in the treatment of this condition [48]. With advancements in the understanding of the genetic basis of leukemia, second-generation BCR-ABL inhibitors such as dasatinib and nilotinib have emerged [49]. These agents have demonstrated greater potency and efficacy in patients who have developed resistance or intolerance to imatinib, thereby expanding the available therapeutic options [50]. However, the emergence of secondary resistance remains a significant challenge, driving the development of third-generation inhibitors. Among them, ponatinib stands out for its effectiveness against mutations that confer resistance to imatinib and second-generation inhibitors, representing a new approach in combating leukemia [51].

Among the latest targeted therapies, Revumenib (Revufoir™), a selective inhibitor, was recently approved by the FDA for the treatment of relapsed or refractory acute myeloid leukemia (AML) with KMT2A rearrangements (MLL1r) [52]. Revumenib acts by disrupting the menin–KMT2A interaction, a critical driver of leukemogenesis in this genetic subtype. Clinical trials have demonstrated its capacity to induce complete remission in heavily pretreated patients [53].

The conventional and advanced treatment strategies available for leukemia and lymphoma are summarized in Table 1.

**Table 1:** Conventional and advanced treatments for leukemia and lymphoma.

Treatment	Mechanism of Action (Summary)
Chemotherapy	Primarily targets rapidly dividing cells by interfering with DNA, RNA, and protein synthesis. Includes classes such as alkylating agents, antimetabolites, topoisomerase inhibitors, and antimicrotubule agents. Induces cell damage leading to apoptosis [54].
Radiation Therapy	Uses ionizing radiation to cause DNA breaks (directly or via free radicals). Triggers cell death and modulates the tumor microenvironment, potentially enhancing immune responses [55].
Targeted Therapy	Focuses on specific molecules or pathways altered in cancer, such as tyrosine kinases, BCL2, or epigenetic regulators. More selective than conventional chemotherapy. Includes monoclonal antibodies and small-molecule inhibitors [56].
Immunotherapy	Enhances or restores the immune system’s ability to recognize and eliminate cancer cells. Includes immune checkpoint inhibitors (e.g., anti-PD-1/PD-L1), CAR-T cells, and BiTEs. Helps reestablish immune surveillance [57,58].
Hematopoietic Stem Cell Transplantation (HSCT)	Replaces bone marrow after myeloablative therapy. Allogeneic HSCT also provides a graft-versus-tumor/leukemia effect, where donor immune cells attack residual malignant cells [59].

**Figure 2:**
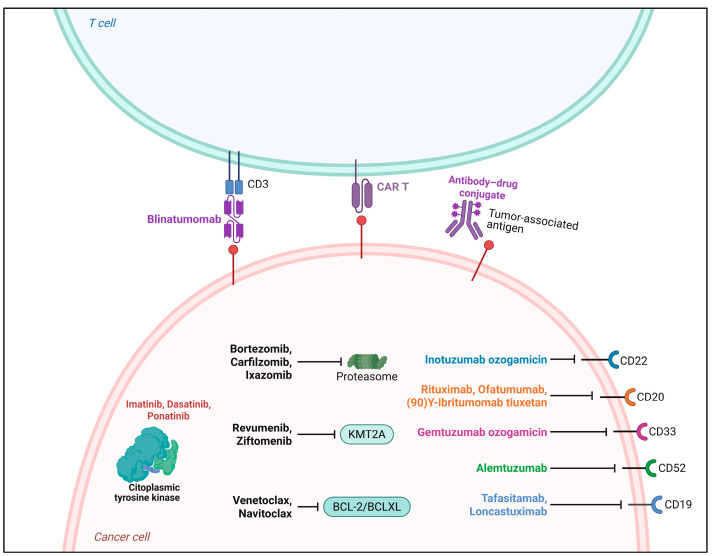
Targeted therapies and immunotherapeutic approaches in the treatment of leukemias, with a focus on acute lymphoblastic leukemia (ALL) and acute myeloid leukemia (AML). The figure illustrates various antineoplastic strategies, including CAR-T cell therapies, bispecific antibodies (such as blinatumomab, which targets CD3 on T cells and CD19 on malignant B cells), antibody–drug conjugates (ADCs), monoclonal antibodies targeting surface antigens (such as CD19, CD20, CD22, CD33, CD52), and small-molecule inhibitors with intracellular targets. Imatinib, dasatinib, and ponatinib inhibit BCR-ABL, a cytoplasmic tyrosine kinase characteristic of Philadelphia chromosome-positive (Ph^+^) ALL. Gemtuzumab ozogamicin, an ADC targeting CD33, is used for CD33-positive AML. The figure also includes therapies under investigation, such as BCL-2/BCL-XL inhibitors, KMT2A inhibitors, and proteasome inhibitors, which have shown promising results in AML. It is important to note that not all targets presented apply equally to both leukemias; for instance, CD33 is highly expressed in AML, while CD19, CD22, and CD20 are specific to ALL. Adapted and corrected from [63]. Created with Biorender.com.

Indeed, immunotherapy has emerged as a groundbreaking therapeutic strategy in the management of leukemia, especially in cases of acute lymphoblastic leukemia (ALL) and acute myeloid leukemia (AML) [60]. One notable example is blinatumomab, a bispecific T-cell engager (BiTE) monoclonal antibody that simultaneously targets CD19 on malignant B cells and CD3 on T cells, thereby redirecting cytotoxic T lymphocytes to eliminate leukemic cells [61]. Blinatumomab has shown remarkable efficacy in patients with relapsed or refractory B-cell acute lymphoblastic leukemia (ALL), achieving high complete remission rates and extended survival outcomes. Similarly, gemtuzumab ozogamicin, an antibody–drug conjugate targeting CD33, has been approved for the treatment of acute myeloid leukemia (AML) in CD33-positive patients, demonstrating significant improvements in response rates and overall survival, particularly among elderly individuals (Figure 2) [62].

Another therapeutic approach involves the use of chimeric antigen receptor T cells (CAR-T), representing a significant innovation in leukemia treatment. These T cells are genetically modified to express specific antigen receptors, allowing them to selectively target cancer cells. CAR-T therapy has shown remarkable results in patients with acute myeloid leukemia (AML), particularly those who experience a relapse after hematopoietic stem cell transplantation. Additionally, CAR-T treatment has proven effective in B-cell acute lymphoblastic leukemia (ALL), achieving impressive complete remission rates [64,65].

Recent therapeutic advances in lymphoma treatment have transformed the clinical approach to this disease, offering patients new perspectives and more effective strategies to combat cancer. One of the key drivers of this progress has been the development and use of monoclonal antibody therapy [66]. Rituximab, for example, has emerged as a crucial element in the treatment of non-Hodgkin B-cell lymphoma. This monoclonal antibody targets CD20, a protein present in malignant B cells, allowing for the selective destruction of these cells by the immune system [67]. The combination of rituximab with chemotherapy has led to impressive response rates and a significant increase in the survival of lymphoma patients. In addition to rituximab, new monoclonal antibodies have been developed and approved for use in lymphoma treatment [68].

Obinutuzumab, for instance, is a second-generation anti-CD20 monoclonal antibody that has demonstrated superiority over rituximab in clinical trials for the treatment of follicular lymphoma. Its enhanced efficacy is attributed to structural modifications that improve antibody-dependent cellular cytotoxicity (ADCC) and direct cell death. Clinical studies, such as the GALLIUM trial, have shown that obinutuzumab, when combined with chemotherapy, leads to longer progression-free survival compared to rituximab-based regimens. As a result, obinutuzumab has become an important therapeutic option, particularly for patients with high-risk disease or those who have not responded adequately to first-line treatments [69].

Additionally, antibody–drug conjugate therapies, such as brentuximab vedotin and polatuzumab vedotin, have demonstrated efficacy in refractory lymphomas, providing an alternative therapeutic option for patients who do not respond to conventional treatments [70].

These therapeutic advances in lymphoma treatment represent a significant shift in the clinical approach, offering patients a broader range of highly effective options and renewed hope for improved outcomes. Monoclonal antibody therapies, including newer, second-generation agents, alongside radionuclide therapy, are transforming the therapeutic landscape. These strategies not only enhance treatment efficacy but also contribute to better tolerability and long-term disease control. Furthermore, there is a growing emphasis on personalized medicine, with treatment increasingly tailored to the genetic profile, disease subtype, and individual characteristics of each patient. This personalized approach aims to maximize therapeutic effectiveness while minimizing adverse effects, ultimately improving both survival and quality of life [71].

## 4. Animal Venoms with Potential Cytotoxic Action Against Leukemia and Lymphoma Cancer Cells

Animal venoms are composed of toxins, enzymes, and bioactive peptides, which play essential roles in prey immobilization and predator deterrence [72]. These compounds demonstrate exceptional specificity toward cellular targets, such as plasma membranes, ion channels, and intracellular signaling pathways, thereby positioning them as promising candidates for the development of innovative anticancer therapeutics. It is also noteworthy that their intrinsic capacity to penetrate various tissues through systemic circulation significantly enhances their suitability for therapeutic applications [73].

Recent investigations have focused on the cytotoxic effects of animal venoms, particularly against leukemia and lymphoma cells [74], and have demonstrated that crude venom from the scorpion *Mesobuthus eupeus* selectively induces cytotoxicity in B-lymphocytes derived from patients with chronic lymphocytic leukemia. The venom exerts its effect by promoting lysosomal and mitochondrial dysfunction, leading to the generation of reactive oxygen species and ultimately triggering cell death. Similarly, identified antimicrobial peptides within scorpion venom that induce pyroptotic cell death—an inflammatory form of programmed cell death mediated by caspase-1—highlighting a novel mechanistic approach for targeting malignant cells [75].

Despite the promising pharmacological potential of venom-derived compounds, only a limited number of such drugs have received approval from the Food and Drug Administration (FDA), totaling six medications currently used in the treatment of conditions including diabetes, chronic pain, hypertension, and coagulation disorders [76]. Recent advancements in biomedical research and bioengineering have contributed to the rational design and structural optimization of venom-derived molecules, resulting in enhanced physicochemical stability, increased target selectivity, and improved safety profiles. These developments have significantly expanded the therapeutic potential of such compounds in the context of cancer treatment [77].

### 4.1. Emerging Therapeutic Agents Derived from Snake Venom

Snake venoms are particularly rich sources of enzymes and peptides with potent biological activities. Among these bioactive compounds, L-amino acid oxidases (LAAOs) and phospholipases A_2_ (PLA_2_s) have garnered significant attention due to their pronounced cytotoxic and apoptotic effects on various cancer cell types, including hematological malignancies. LAAOs, in particular, are flavoenzymes commonly found in snake venoms and are known to contribute substantially to the overall toxicity observed during envenomation [78]. These enzymes exhibit a broad spectrum of biological effects, including cytotoxicity, apoptosis induction, hemorrhagic activity, edema formation, and modulation of platelet aggregation. Additionally, LAAOs display antimicrobial and antiprotozoan activities, underscoring their potential for diverse biotechnological applications [79].

The primary mechanism underlying LAAO-induced cytotoxicity is their enzymatic activity, specifically the oxidative deamination of L-amino acids, which results in the production of hydrogen peroxide (H_2_O_2_), ammonia, and the corresponding α-keto acids. The generation of H_2_O_2_, a potent reactive oxygen species (ROS), induces considerable oxidative stress in and around target cells. This oxidative stress is widely recognized as the principal driver of LAAO-mediated cellular injury, promoting apoptotic and/or necrotic cell death [80,81]. Supporting this mechanism, numerous studies have shown that the cytotoxic and apoptotic effects exerted by LAAOs are markedly diminished or completely abrogated upon treatment with catalase, an enzyme responsible for degrading H_2_O_2_, thus confirming the pivotal role of this ROS in mediating LAAO-induced cell death [82].

Various LAAOs isolated from snake venoms have demonstrated selective cytotoxic and pro-apoptotic effects on hematological cancer cells, further underscoring their therapeutic potential. LAAO from *Agkistrodon* spp. (Korean snake) induced apoptosis in vascular endothelial and murine L1210 leukemia cells, characterized by DNA laddering and attributed to localized H_2_O_2_ production following cell surface binding, although the apoptotic pathway appeared distinct from that triggered by exogenous H_2_O_2_ [83].

MipLAAO from *Micrurus mipartitus*’s venom selectively induced apoptosis in Jurkat T-ALL cells while sparing normal peripheral blood lymphocytes, a favorable property for therapeutic use. This activity was associated with increased intracellular oxidative stress, the upregulation of PUMA and p53, the phosphorylation of c-JUN, and caspase-3 activation [84].

BatroxLAAO from *Bothrops atrox*’s venom showed cytotoxicity toward HL-60 and Jurkat cells, inducing both apoptosis and necrosis in a dose-dependent manner, with reduced effects in the presence of catalase, confirming H_2_O_2_’s dependence. Similarly, BpirLAAO from *Bothrops pirajai* activated caspases 3, 8, and 9 and induced apoptosis in HL-60 and HL-60.Bcr-Abl cells, suggesting its potential efficacy against tyrosine kinase inhibitor-resistant leukemias. An LAAO from *Agkistrodon halys* also induced apoptosis in multiple leukemia cell lines; however, catalase only partially restored viability, indicating potential H_2_O_2_-independent mechanisms. Apoxin I from *Crotalus atrox* required H_2_O_2_ for its activity and was inhibited by the membrane antioxidant, suggesting the involvement of membrane lipid peroxidation [85]. VB-LAAO from *Vipera berus berus* displayed dose-dependent cytotoxicity in K562 CML cells, shifting from apoptosis to necrosis at higher concentrations, consistent with the hypothesis that oxidative stress intensity dictates the mode of cell death. CR-LAAO from *Calloselasma rhodostoma* initially induced necrosis in Jurkat cells, but catalase treatment shifted the response toward apoptosis, suggesting that high H_2_O_2_ levels may suppress apoptotic pathways [86]. Moreover, CR-LAAO modulated the expression of multiple microRNAs (e.g., miR-145, miR-26a, miR-142-3p, miR-21, miR-130a, miR-146a) and apoptosis-regulating proteins (e.g., Bid, Bim, Bcl-2, cIAP2, c-FLIP, Mcl-1) in Bcr-Abl-positive CML cells, indicating a complex regulation of apoptotic signaling beyond oxidative damage alone [87].

Phospholipases A_2_ (PLA_2_s) are enzymes that hydrolyze the ester bond at the sn-2 position of membrane phospholipids, liberating lysophospholipids and free fatty acids, notably, arachidonic acid, a precursor in pro-inflammatory signaling cascades [88]. These enzymes are small, stable proteins (13–15 kDa) that are highly abundant in snake venoms and contribute to a diverse spectrum of toxic and pharmacological effects, including myotoxicity, neurotoxicity, inflammation, anticoagulant activity, and cytotoxicity [89,90]. PLA_2_s are broadly categorized based on the amino acid residue at position 49, with Asp49 PLA_2_s being catalytically active and responsible for enzymatic hydrolysis, while Lys49 variants exhibit negligible catalytic activity but retain significant biological activity, including strong myotoxic and cytotoxic properties [91]. Among the phospholipases A_2_ (PLA_2_s) studied for their antileukemic properties, MjTX-I, an Asp49 PLA_2_ isolated from *Bothrops moojeni*’s venom, demonstrated pronounced cytotoxic activity against chronic myeloid leukemia (CML) cells, including both imatinib-sensitive (K562-S) and imatinib-resistant (K562-R) lines, while exhibiting minimal toxicity toward non-tumor cells such as HEK-293 and peripheral blood mononuclear cells (PBMCs) [92].The selective pro-apoptotic action of MjTX-I was associated with the activation of caspase-8, caspase-9, and caspase-3, the cleavage of poly (ADP-ribose) polymerase (PARP), the downregulation of the anti-apoptotic protein BCL-2, and the upregulation of the pro-apoptotic protein BAD [93].

This suggests that MjTX-I induces apoptosis primarily through the mitochondrial pathway, circumventing mechanisms that confer resistance to tyrosine kinase inhibitors, such as mutations in the Bcr-Abl gene or the activation of alternative survival pathways, thereby highlighting its potential role as a second-line or combinatorial therapeutic agent in CML treatment [94]. In contrast, BJ-PLA_2_-I, an acidic Asp49 PLA_2_ from *Bothrops jararaca* venom, despite exhibiting high catalytic activity, showed only modest cytotoxic effects on HL-60 leukemia cells and PBMCs, with significant viability reduction observed only at high concentrations (160 µg/mL). Notably, BJ-PLA_2_-I was a potent pro-inflammatory agent in vivo, promoting neutrophil migration and elevating levels of IL-6, IL-1β, and prostaglandin E2 (PGE2), thereby highlighting its role in venom-induced inflammation rather than selective antitumor activity [95].

Integrin α_5_β_1_ (VLA-5), a primary receptor for fibronectin, has been shown to be functionally important in Philadelphia chromosome-positive acute lymphoblastic leukemia (Ph+ ALL) [96]. Its expression is upregulated in Ph+ ALL cells (SUP-B15 cell line) under stress conditions such as serum deprivation. Functionally, α_5_β_1_ mediates the adhesion of these cells to fibronectin and contributes to their survival and resistance to apoptosis. Binding studies with disintegrins also indicate that α_5_β_1_ is a relevant target in chronic myeloid leukemia (CML–K562), T-cell acute lymphoblastic leukemia (T-ALL–Jurkat), lymphoblastoid cells (JY), acute promyelocytic leukemia (APL–HL-60), and acute myeloid leukemia (AML–THP-1) cell lines [97].

Integrin α_v_β_3_, the disintegrin Moojecin, isolated from *Bothrops moojeni*’s venom, has been shown to interact more strongly with α_v_β_3_ than with α_5_β_1_ in assays using AML cells (HL-60 and THP-1) [98]. Although more extensively studied in solid tumors and angiogenesis, its presence as a target in AML is noteworthy. Antibodies targeting α_v_β_3_ (such as MEDI-522) have also been evaluated in solid tumor models [99].

A wide range of venom-derived disintegrins (such as EC3, EC6, Echistatin, EMF-10, EO5, Jerdostatin, Lebestatin, Obtustatin, VA6, VB7, Viperistatin, and VLO5) have demonstrated the ability to inhibit the adhesion of leukemia (K562, Jurkat) and lymphoblastoid (JY) cell lines to extracellular matrix (ECM) components such as fibronectin, or in general, adhesion assays [100]. Specifically, in Ph+ ALL, both a generic disintegrin peptide and an anti-α_5_ inhibitory antibody were able to block adhesion to fibronectin [101]. In contrast, the disintegrin-like domains of endogenous ADAMs (e.g., ADAM28, ADAM7, ADAM33) can promote the adhesion of transfected Jurkat or K562 cells via α_4_β_1_, α_4_β_7_, or α_9_β_1_ integrins, highlighting the complexity of these interactions [102].

In addition to LAAOs and PLA_2_s, snake venom lectins (SVLs) have emerged as promising candidates in the search for novel therapeutic agents against hematological malignancies. Lectins isolated from *Bothrops* species, particularly BJcuL (Bothrops jararacussu lectin) and BJL (Bothrops jararaca lectin), exhibit high affinity for specific glycan residues abundantly expressed on the surface of leukemic cells [103,104]. Upon binding to these glycoconjugates, SVLs can trigger a cascade of intracellular events leading to apoptosis. One of the primary mechanisms involves mitochondrial membrane depolarization, with subsequent cytochrome c release and the activation of caspases, particularly caspase-9 and caspase-3, characteristic of the intrinsic apoptotic pathway [105]. Additionally, BJcuL has been shown to increase intracellular reactive oxygen species (ROS) levels, contributing to oxidative stress and amplifying apoptotic signaling [106].

In human leukemia HL-60 cells, BJcuL exposure resulted in the upregulation of pro-apoptotic Bax, downregulation of anti-apoptotic Bcl-2, activation of caspase-3, and PARP cleavage, collectively confirming the activation of the mitochondrial apoptosis pathway [107].

Crotamine is a small polypeptide originally isolated from the venom of the South American rattlesnake *Crotalus durissus terrificus*. It induces apoptosis in HL-60 cells, a process that appears to be associated with changes in mitochondrial membrane permeability and cytochrome c release [108]. Interestingly, an in vivo study using an HL-60 xenograft model reported that crotamine exhibited lower tumor growth inhibitory activity compared to solid tumor models, suggesting a potential differential efficacy depending on tumor type or microenvironment [109]. A crotamine-like peptide (CLP), isolated from the venom of *Crotalus oreganus helleri*, also demonstrated dose-dependent cytotoxicity in that cell line [110]. In addition, native crotamine induces cell death in K562 cells through mechanisms involving mitochondrial collapse, cytochrome c release, and caspase-3 activation, ultimately leading to apoptosis and autophagy [111].

### 4.2. Anticancer Effects of Bee-Derived Compounds

Bee venom (BV), secreted by Apis mellifera, is a complex natural secretion historically used in traditional medicine (apitherapy) and increasingly recognized for its pharmacological properties, particularly its anticancer potential [112,113,114]. BV is composed of a synergistic blend of bioactive compounds, including enzymes such as phospholipase A2 (PLA2) and hyaluronidase; peptides like melittin (MEL), apamin, and mast cell degranulating (MCD) peptide; along with amines and other small molecules [115].

Melittin (MEL) is the principal peptide component of bee venom (BV), accounting for approximately 40–60% of its dry weight [116]. It is a small, 26-amino acid amphipathic peptide with well-documented biological activity, particularly for its membrane-disruptive properties [117]. The primary mechanism of MEL involves direct interaction with cell membranes, where it inserts into the phospholipid bilayer, forming pores or channels that compromise membrane integrity [118]. This disruption results in the leakage of ions and intracellular components, ultimately causing cell lysis [119].

Interestingly, MEL appears to display a certain selectivity for cancer cell membranes, likely due to variations in membrane fluidity, surface charge, lipid composition, or membrane potential between cancerous and normal cells [120]. This nonspecific yet potent mechanism of action offers a significant advantage in combating therapeutic resistance, a frequent limitation of conventional targeted therapies, since it may be more difficult for cancer cells to escape through single protein alterations [121]. Nonetheless, MEL’s broad lytic activity also raises concerns about its systemic toxicity [122].

Several studies have demonstrated the antitumor effects of MEL against various leukemia and lymphoma cell lines, including CCRF-CEM (acute lymphoblastic leukemia), K-562 (chronic myeloid leukemia), U937 (histiocytic lymphoma/leukemia), and HL-60 (acute promyelocytic leukemia) [123].

The specific mechanisms by which melittin induces the death of hematological cancer cells include the induction of apoptosis in multiple leukemia cell lines [124]. This is evidenced by the disruption of mitochondrial membrane potential, Annexin V binding (indicating phosphatidylserine externalization), and the activation of caspases 3 and 7 in CCRF-CEM and K-562 cells [125]. In MOLT-4 cells, melittin-induced apoptosis appears to occur through a caspase-3-independent pathway. Moreover, melittin may enhance the cytotoxicity of other apoptosis-inducing agents, such as TNF, in leukemic cells—possibly via phospholipase A_2_ (PLA_2_) activation [126,127].

In addition to apoptosis, melittin can induce necrosis or direct cell lysis, particularly at higher concentrations, due to its pore-forming ability in the plasma membrane [128]. In Burkitt lymphoma cells, melittin-induced pore formation has been shown to exert effects similar to those of the complement system’s membrane attack complex [129].

Currently, the therapeutic application of melittin has been extensively investigated, with a focus on selective delivery strategies such as the use of nanoparticles, liposomes, antibody-conjugation systems, and tumor-targeting peptide vectors for hematological malignancies [130]. Structural modifications, such as PEGylation or the use of D-amino acids, are also being explored to reduce toxicity. These approaches aim to preserve melittin’s antitumor effect while minimizing its cytotoxicity to healthy cells and expanding its therapeutic window [131]. Additionally, recent studies have shown that melittin can act synergistically with conventional chemotherapeutics (such as cisplatin in resistant Hodgkin lymphoma cells) or tyrosine kinase inhibitors, suggesting its potential as an adjuvant in combination therapy regimens [132].

### 4.3. Scorpion Venom Peptides and Components Against Leukemia and Lymphoma

Scorpion venoms (SVs) are a rich source of pharmacologically active compounds, especially peptides that often contain stabilizing disulfide bridges [133,134]. Much like bee venom, scorpion venom has been traditionally used in various medicinal practices [135,136]. Recent scientific research has highlighted its potent effects against a range of cancer types, including leukemia and lymphoma, demonstrating its potential as an anticancer agent [137].

For example, Bengalin, a protein purified from the venom of the Indian black scorpion (*Heterometrus bengalensis*), exhibits potent pro-apoptotic activity against human leukemic cell lines U937 and K562. Its mechanism involves triggering a mitochondrial death cascade, which results in the loss of mitochondrial membrane potential, the activation of initiator caspase-9 and executioner caspase-3, and the subsequent cleavage of PARP [138].

Scorpion Venom Component III (SVCIII), a peptide fraction (~70–80 kDa) isolated from the venom of *Buthus martensii Karsch* (BmK), inhibits the proliferation of human leukemia cell lines THP-1 (acute monocytic leukemia) and Jurkat (T lymphoma) [139]. Unlike direct cytotoxic agents, SVCIII induces cell cycle arrest in the G1 phase by downregulating the key cell cycle regulator cyclin D1. Moreover, it inhibits the NF-κB signaling pathway, which is critical for survival and proliferation in hematopoietic malignancies. Specifically, SVCIII prevents the phosphorylation and degradation of IκBα, thus inhibiting the nuclear translocation of the p65 NF-κB subunit. This mechanism offers a distinct approach compared to direct cytotoxic agents, potentially leading to cell cycle arrest or differentiation rather than immediate cell death, which could be advantageous in therapeutic contexts or combination regimens [140,141].

Other components of *Buthus martensii Karsch* venom, including crude venom and isolated peptides, have also shown activity against cancer cells. For instance, crude venom induces cell cycle arrest and apoptosis in human lymphoma cells (Raji, Jurkat), while the toxin BmKKx2 causes G1 arrest and differentiation-dependent apoptosis in K562 CML cells. Additionally, the peptide BmKn-2 induces apoptosis in various cancer types via caspase activation and modulation of the p53/Bax/Bcl-2 axis [142].

Another promising compound, Smp24, a cationic antimicrobial peptide from *Scorpio Maurus palmatus* venom, has exhibited cytotoxicity against acute leukemia cell lines KG1-a and CCRF-CEM [143]. Although its detailed mechanism in leukemia cells requires further study, investigations in HepG2 liver cancer cells revealed that Smp24 exerted a multi-pronged attack, including the disruption of cell membranes, mitochondrial dysfunction leading to apoptosis, cell cycle arrest, and the induction of autophagy [144].

### 4.4. Cone-Snail-Venom-Derived Conotoxins Against Leukemia and Lymphoma Cells

The investigation into the effects of conotoxins on hematological malignancies has revealed promising results, particularly in leukemia cells. Specific studies have explored the potential of *Conus* venoms and conotoxin fractions. The crude venom of the vermivorous cone snail *Conus vexillum*, collected from the Red Sea, has shown concentration-dependent cytotoxic effects against Ehrlich ascites carcinoma (EAC) cells in in vitro studies. More notably, the crude venom of *Conus textile* and, specifically, its fraction B, were found to significantly reduce viability in B lymphocytes from chronic lymphocytic leukemia (CLL) patients. That study highlighted a promising candidate for inducing apoptosis in CLL patients [145,146].

Elucidation of these mechanisms of action is essential for understanding the therapeutic potential of *Conus* venoms in cancer treatment. The venom of *Conus vexillum* has been shown to induce significant oxidative stress in EAC cells, as demonstrated by increased lipid peroxidation, elevated protein carbonyl content, and the accumulation of reactive nitrogen intermediates [147]. These effects were accompanied by a marked reduction in the activity of key antioxidant defense enzymes, such as catalase and superoxide dismutase, as well as a decrease in total antioxidant capacity. These findings suggest that the cytotoxicity exerted by *C. vexillum* venom is mediated, at least in part, by oxidative stress pathways. In the case of fraction B from *Conus textile* venom, its cytotoxic effects on chronic lymphocytic leukemia (CLL) B lymphocytes were associated with a cascade of molecular events typically observed during programmed cell death. This included a significant increase in intracellular reactive oxygen species (ROS) levels, a collapse of mitochondrial membrane potential (MMP), lysosomal membrane destabilization, and the subsequent activation of caspase-3, a key executioner of the apoptotic process [148].

Although there is compelling evidence for leukemia and other solid tumors (e.g., malignant pleural mesothelioma, glioma) [149,150], detailed and specific studies on the activity of conotoxins against lymphoma cell lines are notably underrepresented in the available literature. Nevertheless, the general mechanisms of action, such as the modulation of ion channels (e.g., HERG potassium channels, sodium and calcium channels) and nicotinic acetylcholine receptors (nAChRs), are broadly relevant to various cancer types, including lymphomas, which often exhibit dysregulated signaling pathways [151,152].

A variety of animal-venom-derived compounds have demonstrated cytotoxic and antitumor activities against leukemia and lymphoma cell lines. These bioactive molecules, including L-amino acid oxidases, phospholipases A_2_, disintegrins, and various peptides from scorpions, bees, and toads, exert their effects through diverse mechanisms such as apoptosis induction, oxidative stress, membrane disruption, and cell cycle arrest.

**Figure 3:**
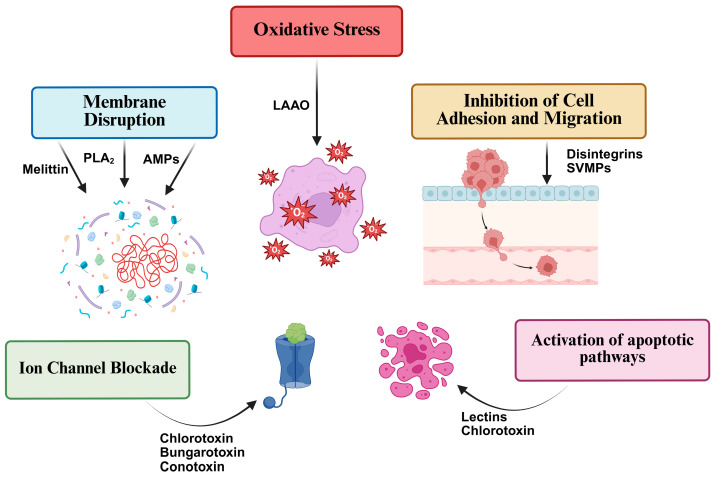
Schematic representation of the main antitumor mechanisms induced by animal-venom-derived molecules in leukemia and lymphoma cells. These include the following: Membrane disruption, caused by peptides such as melittin and phospholipases A_2_ (PLA_2_s), leading to increased membrane permeability and cell lysis; ion channel blockade, triggered by neurotoxins like chlorotoxin and conotoxins, disturbing ion homeostasis and activating cell death pathways; the induction of oxidative stress, mainly mediated by L-amino acid oxidases (LAAOs), promoting excessive production of reactive oxygen species (ROS) and oxidative damage to cellular macromolecules; the inhibition of cell adhesion and migration, observed with disintegrins and snake venom metalloproteinases (SVMPs), which interfere with integrin-mediated cell–extracellular matrix interactions and reduce tumor cell invasiveness; and the activation of apoptotic pathways, involving both intrinsic (mitochondrial) and extrinsic (death-receptor-mediated) routes, often characterized by caspase activation, mitochondrial membrane permeabilization, and the modulation of apoptotic regulators such as Bcl-2 family proteins. Collectively, these mechanisms highlight the therapeutic potential of animal venom components as antitumor agents targeting leukemia and lymphoma cells. Created with Biorender.com.

To visually summarize the main molecular mechanisms through which these venom components exert their antitumor effects against leukemia and lymphoma cells, a schematic overview is presented in Figure 3 below.

Crude venoms also demonstrated antineoplastic properties against hematological malignancies. Studies targeting leukemias and lymphomas have shown that venoms can modulate key signaling pathways, induce apoptotic cell death, and interfere with the cell cycle of malignant cells. These insights hold significant promise for the advancement of more precise and efficacious therapeutic strategies, potentially mitigating the adverse effects associated with conventional cancer treatments [153,154]. To provide a systematic overview of research in this field, Table 2 summarizes key animal species whose crude venoms exhibit cytotoxic activity against leukemia and lymphoma cells. The table includes details on bioactive compounds, their proposed mechanisms of action, and supporting scientific references, offering a robust foundation for future investigations and the development of venom-based therapeutic interventions.

## 5. Venoms and Toxins: A Promising Source for the Development of New Drugs

The exploration of naturally derived bioactive compounds has been a cornerstone of pharmaceutical innovation, with venoms and toxins emerging as particularly rich sources of therapeutic molecules. Many of the studies analyzed here highlight the evolutionary refinement of venomous secretions, which has led to the production of a vast repertoire of peptides, enzymes, and small molecules with highly specific biological functions, many of which have shown significant potential in drug development [179]. As traditional drug discovery methods face challenges such as resistance mechanisms in pathogens and cancer cells, venom-based compounds offer an alternative avenue for identifying novel pharmacological agents with high specificity and potency [180].

One of the key advantages of venom-derived molecules is their ability to interact precisely with molecular targets, often at nanomolar concentrations. These interactions frequently involve ion channels, G protein-coupled receptors, and enzymatic pathways that play crucial roles in disease pathophysiology [181,182]. For instance, toxins from scorpions, snakes, and arachnids have been found to modulate sodium and potassium channels, making them valuable candidates for treating neurological disorders, cardiac arrhythmias, and chronic pain [183]. Additionally, enzymatic components, such as phospholipases and metalloproteases, have shown promise in modulating inflammatory responses, immune system activity, and tumor progression [184].

Recent advancements in venom research have led to a deeper understanding of how these bioactive compounds exert their effects at the cellular and molecular levels [185]. Beyond their direct cytotoxicity against cancer cells, some venom-derived peptides have demonstrated immunomodulatory properties, which could enhance existing immunotherapies [186]. For example, bee venom components, particularly melittin, have been studied for their ability to enhance dendritic cell activation and promote an immune response against tumor cells [187]. Similarly, certain snake venom proteins have been shown to suppress angiogenesis, thereby limiting the blood supply to tumors and inhibiting their growth [188].

Despite these promising findings, one of the major challenges in venom-based drug discovery is the isolation and characterization of bioactive molecules from complex venom mixtures. Modern proteomic and transcriptomic technologies have facilitated the identification of novel peptides, while synthetic biology approaches have enabled their large-scale production [189]. In particular, recombinant expression systems have been employed to produce venom-derived peptides with optimized stability and reduced immunogenicity, making them more suitable for therapeutic applications. Moreover, chemical modifications and nanoformulations are being explored to improve the pharmacokinetics of venom-based compounds, enhancing their bioavailability and reducing potential side effects [190].

The potential applications of venom-derived molecules extend beyond oncology and neurology, encompassing areas such as infectious diseases, metabolic disorders, and even regenerative medicine [191]. Studies on antimicrobial peptides from scorpion venom, for instance, have revealed their ability to target drug-resistant bacterial strains, positioning them as alternatives to conventional antibiotics [192]. Likewise, components from amphibian venoms have been explored for their regenerative properties, particularly in promoting wound healing and tissue repair [193].

As venom research progresses, interdisciplinary collaboration between toxicologists, pharmacologists, and biomedical engineers will be crucial in translating these discoveries into clinically viable treatments [194,195]. The integration of computational modeling and artificial intelligence is also expected to accelerate drug discovery by predicting bioactivity and optimizing molecular modifications for improved therapeutic efficacy [196]. Given the immense biodiversity of venomous species, of which only a fraction has been studied in detail, it is likely that many more pharmacologically relevant compounds remain undiscovered [197].

## 6. Conclusions

Since the discovery of cancer, no treatment has proven to be entirely effective in selectively eliminating tumor cells without damaging healthy tissues. This historical limitation highlights the ongoing and urgent need for antitumor agents that are both more selective and less toxic. Chemotherapy and radiotherapy, although widely used, often lead to severe side effects due to their lack of specificity, significantly compromising patients’ quality of life. In this context, the bioprospecting of venoms from venomous animals emerges as an innovative and promising approach for the development of more effective and targeted therapies, especially against hematological malignancies such as leukemia and lymphoma. Venoms from snakes, scorpions, spiders, and other venomous organisms contain complex mixtures of bioactive molecules that interact with biological systems in highly specific ways. Several studies have demonstrated that proteins, peptides, and other components derived from these venoms can inhibit tumor cell proliferation, induce apoptosis, and interfere with critical survival signaling pathways, such as NF-κB and PI3K/Akt. Although preclinical findings are promising, further research is essential to assess the toxicity, pharmacokinetics, and delivery systems of these compounds. Interdisciplinary collaboration among biologists, chemists, pharmaceutical scientists, and clinicians will be crucial to translate these findings into viable and safe oncological therapies.

With continued research, standardization, and rigorous clinical testing, venom-derived compounds may become a valuable component of the modern oncological arsenal, offering more effective and selective treatments with fewer adverse effects for cancer patients.

Despite the promising results reported for various animal venom components, it is important to emphasize that most available studies were conducted in vitro, predominantly using solid-tumor-derived cell lines. This represents a significant limitation when extrapolating findings to hematological malignancies such as leukemia and lymphoma. Additionally, few studies have assessed the effects of venom components on normal, healthy hematopoietic cells, posing concerns regarding therapeutic selectivity and safety. The scarcity of in vivo preclinical models further restricts the current understanding of pharmacodynamics, biodistribution, toxicity, and potential off-target effects. Therefore, future research should prioritize the development of experimental models specific to hematological cancers, with a focus on validating in vitro observations and ensuring clinical relevance for non-solid tumors.

## Figures and Tables

**Table 2:** Animal-derived venoms with anti-leukemia/lymphoma activities.

Species	Lineage	Action Factor	Effect	Activity
*Apis mellifera*	CCRF-CEM (ALL), K-562 (CML), U937, HL-60	Melittin	Induces mitochondrial apoptosis (caspase-3/7), downregulates ERK/Akt and NF-κB pathways, and modulates Bcl-2, c-MYC, CDK4, among others	Cytotoxic, pro-apoptotic, and intracellular signaling modulator [155,156]
*Bothrops erytromelas, Bothrops jararaca, Bothrops alternatus*	Leukemia–K562	X	Reduction of cell viability and proliferation	Morphological alterations, plasma membrane rupture, presence of pyknotic cells, increased membrane permeability, loss of mitochondrial function, higher total DNA damage index, reduction in transcript levels of CCN1, CCNH, CDK2, CDK1, and BCR-ABL1, increased expression of cell cycle inhibitors CDKN1A and WEE1, reduced gene expression of CCNB1, CCNH, CDK1, and BCR-ABL1 [157]
*Bothrops jararacussu*	Acute promyelocytic leukemia–HL-60	BthTX-I	Reduction of cell viability	Induction of necrosis and apoptosis, 75% to 90% cytotoxicity [158]
*Bothrops mattogrossensis*	Acute T-cell leukemia–JURKAT	BmatTX-I e BmatTX-II	Apoptosis	Changes in the cell membrane, catalytic activity-independent cytotoxic activity [159].
*Bothrops moojeni*	Chronic myeloid leukemia–K562-S and K562-R Bcr-Abl +	MjTX-I	Reduction in cell viability by up to 65%	Increase from 45.5% to 62% in hypodiploid nuclei, high levels of cell death, reduced expression levels of pro-caspase 3, and increased expression levels of caspase 9 in K562-S lineage, reduced expression of pro-caspase 3, 8, and 9, and higher levels of cleaved PARP in K562-R lineage, reduced level and expression of the anti-apoptotic gene BCL-2, BAD, BAX, CLL-XL, and c-FLIP in K562-S, and increased expression levels of the pro-apoptotic gene BAD in K562-R [160]
*Bothrops brazili*	Acute T-cell leukemia–JURKAT	MTX-I e MTX-II	Likely induction of apoptosis	Independent of catalytic activity [160]
*Bothrops moojeni*	Acute T-cell leukemia–JURKAT	MjTX-II	X	Independent of catalytic activity, induction of apoptosis [161]
*Crotalus oreganus helleri*	Chronic myeloid leukemia–K-562	CLP	Reduction in cell viability	Induction of apoptosis and necrosis resulting from increased lysosomal membrane permeability, mitochondrial swelling [162]
*Bothrops pauloensis*	Acute T-cell leukemia–JURKAT TIB-152™	Bp-LAAO	Cell death	Dose-dependent cytotoxicity, inhibition of tumor growth [163]
*Crotalus atrox*	Promyelocytic leukemia–HL-60	Apoxin I	X	Morphological cellular changes, induction of chromatin condensation and segregation, induction of apoptosis [164]
*Crotalus atrox*	T-cell lymphoma–S-49	X	X	X [164]
*Crotalus durissus terrificus*	Murine erythroleukemia and chronic myeloid leukemia–K-562	CTX	Reduction in cell viability	Cell death and lysis (40%), collapse of mitochondrial membrane potential, autophagy, apoptosis, vacuolization and mitochondrial swelling, nuclear condensation, pyknosis, organelle loss, significant reductions in cytochrome c levels in the cytosol, cell membrane rupture [165]
*Bothrops jararaca*	Promyelocytic leukemia–HL-60	BJ-PLA 2 -I	Reduction in cell viability	Low cytotoxicity (70% to 80%) [166]
*Bothrops jararacussu*	Acute T-cell leukemia–JURKAT	BthA-I-PLA 2	Cell death	Induction of apoptosis [166].
*Bothrops atrox*	HL-60 (APL), Jurkat (T-ALL)	BatroxLAAO	H_2_O_2_ induces cytotoxicity through oxidative stress, activates apoptosis via caspases-3 and -9, and causes cell cycle arrest at the G0/G1 phase, inhibiting cell proliferation	Its main activity is pro-oxidant, acting as a generator of reactive oxygen species (ROS), which trigger these cellular responses [167]
*Calloselasma rhodostoma*	Jurkat (T-ALL), Bcr-Abl+ CML cells	CR-LAAO	H_2_O_2_ induces the transition from necrosis to apoptosis and modulates apoptomiRs and apoptosis-regulating proteins, such as Bcl-2, in chronic myeloid leukemia (CML) cells	It acts as a pro-oxidant, inducing oxidative stress and modulating apoptotic pathways, including microRNAs and apoptosis-regulating proteins [168,169]
*Androctonus aeneas (Scorpion–North American)*	JURKAT	Bmk AGAP	X	Blocks the action of lymphoma and glioma CCL-86 lineage and T-lymphocytes derived from adult T-cell leukemia/lymphoma [170]
*H. bengalensis Kochveneno*	Leukemic cells U937 and K562.	x	Inhibition of cell proliferation in U937 and K562 occurred through apoptosis, evidenced by damaged nuclei and cell cycle arrest in the sub G1 phase	Increased DNA fragmentation and also reduced telomerase activity [171]
*Leiurus quinquestriatus*	B-cell lymphoma-2	x	x	Immunohistochemical results showed a decrease in the expression of molecular markers such as Ki-67, nuclear factor kappa-B, cyclooxygenase-2, B-cell lymphoma-2, and vascular endothelial growth factor in animals treated with venom [172]
*Jordanian honeybee (JCBV)*	Leukemic K562	Melittin	Cell death	Late apoptotic cell death with moderate cell cycle arrest [173]
*Bufo melanostictus*	Leukemic K562, U937, ML1 e HL60	Bufalina	Cell differentiation	Exhibited a potent differentiation-inducing activity [159]
*Bufo melanostictus*	Leukemic THP-1 and MOLT-3	Bufalina	Cell death	Induced apoptosis [159]
*Aetobatus narinari*	Leukemic Jurkat E6-1	SRV	Significant growth inhibitory effects in cells	Induced apoptosis and necrosis [174]
*Micrurus mipartitus*	Jurkat (T-ALL)	MipLAAO	Induces apoptosis via caspase-3, p53, and PUMA	Pro-oxidant and apoptosis inducer [175]
*Heterometrus bengalensis*	U937, K562 (CML)	Bengalin	Induces mitochondrial apoptosis with caspase-3/9 activation and PARP cleavage	Pro-apoptotic/mitochondrial apoptosis inducer [176]
*Buthus martensii Karsch*	THP-1 (Monocytic Leukemia), Jurkat (T Lymphoma)	SVCIII	Causes G1 cell cycle arrest (by downregulating cyclin D1) and inhibits the NF-κB pathway (by reducing IκBα degradation and p65 nuclear translocation)	Antiproliferative and NF-κB signaling inhibitor [177]
*Maurus palmatus*	KG1-a (AML), CCRF-CEM (ALL)	Smp24	Induces cytotoxicity through membrane disruption and mitochondrial dysfunction, leading to apoptosis, cell cycle arrest, and autophagy	Cytotoxic, pro-apoptotic, and cell stress inducer [178]

## Data Availability

The data presented in this study are openly available in public repositories and indexed databases. All original information was obtained from peer-reviewed articles accessed through PubMed, Scopus, Web of Science, and ScienceDirect. No new data were generated in this review.
